# Team-based learning pedagogy enhances the quality of Chinese pharmacy education: a systematic review and meta-analysis

**DOI:** 10.1186/s12909-019-1724-6

**Published:** 2019-07-29

**Authors:** Bingchen Lang, Lingli Zhang, Yunzhu Lin, Lu Han, Chuan Zhang, Yantao Liu

**Affiliations:** 1 Department of Pharmacy, West China Second University Hospital, Sichuan University, No. 20, Section 3, Renmin Nanlu, Chengdu, 610041 Sichuan China; 2 Key Laboratory of Birth Defects and Related Diseases of Women and Children (Sichuan University), Ministry of Education, Chengdu, China; 3 Evidence-Based Pharmacy Center, West China Second University Hospital, Sichuan University, Chengdu, China

**Keywords:** Team-based learning, Lecture-based learning, Chinese pharmacy education, Meta-analysis

## Abstract

**Background:**

Recent years have witnessed the wide application of team-based learning(TBL) pedagogy in Chinese pharmacy education. However, the relevant systematic review evaluating the effects of such new pedagogical approach has not been established. The present study was designed to examine systematically the effect of using TBL approach in pharmacy education in China.

**Methods:**

Six databases were searched from the inception to January 2019. The studies reporting the performance of pharmacy students in Chinese university or college receiving TBL pedagogy compared to those receiving traditional lecture-based learning (LBL) were enrolled to be analyzed. Scores of the objective theoretical test were considered as the primary outcome, and the results from questionnaires about the number of students who approved the effects of TBL pedagogy on improving their learning enthusiasm, self-study ability, thinking ability, and communication skills were considered as the secondary outcome. A meta-analysis was conducted following the guidelines of the Cochrane Reviewer’s Handbook and the Preferred Reporting Items for Systematic Reviews and Meta Analyses statement.

**Results:**

A total of 1271 students in 12 studies published from 2013 to 2018 were enrolled in present analysis. Compared with traditional LBL pedagogy, TBL pedagogy exhibited more effectiveness in developing the objective tests scores of pharmacy students from both universities (SMD = 1.69, 95% CI [1.10, 2.28], *p* < 0.00001) and colleges (SMD = 4.37, 95% CI [1.33, 7.40], *p* < 0.00001), and such pedagogy applied well in experiments-oriented courses (SMD = 2.14, 95% CI [0.86, 3.43], *p* < 0.00001) and theory-oriented courses (SMD = 2.77, 95% CI [1.41, 4.14], *p* < 0.00001). In addition, it developed students’ learning enthusiasm, students’ self-study ability, thinking ability, and enhanced students’ communication skills.

**Conclusions:**

TBL pedagogy has developed rapidly and applied widely in Chinese pharmacy education during the last decade. The results indicated that such novel pedagogy is compatible with the present situation of Chinese pharmacy education. And it could be considered as an effective method to enhance both the theoretical test scores and various abilities of Chinese pharmacy students.

**Electronic supplementary material:**

The online version of this article (10.1186/s12909-019-1724-6) contains supplementary material, which is available to authorized users.

## Background

The past few decades have witnessed the dramatic developments and reforms in teaching approaches around the world. Compared with the traditional lecture-based learning (LBL), these innovated teaching methods are more emphatic in the students’ innovative consciousness, active communication and creative ability. As one innovative and interactive pedagogical approach, Team-based learning (TBL) expanded beyond its initial business domain into medical school around the world [1–7]. The introduction of such pedagogical approach enlightens students’ learning motivation and tendency to think autonomously. More importantly, it creates a positive atmosphere in classroom for students to communicate mutually and to solve the problems cooperatively.

However, owing to imbalance and shortage of teaching resources, and the restriction of traditional teaching conceptions, LBL is still the mainstream in the Chinese medical education system. Thus, the long period of simplex and cramming teaching brought Chinese students introverted, quiet personality, and passive study habits. Fortunately, although unknown and hardship existed constantly, the reformation and innovation of Chinese medical education has been proceeding in last ten years [8].

As a latecomer, TBL pedagogy has emerged and developed rapidly during the recent decades in medical education of China. It has been applied in various universities and colleges, and its effectiveness on improving the educational quality has been proved [9]. Compared with the medical curricula, pharmacy teaching in China places emphasis on the theoretical tests of factual knowledge and the performance of laboratory practice. But shortage of educational materials (classrooms and equipment) and enormous number of students made the classroom still teacher-oriented [10]. Notably, TBL approach provides the advantage that ensuring the effectiveness of small-group learning with high student faculty ratios [11], which considerably fits for the understaffed condition of pharmacy education in China. Recently, increasing studies have revealed the efficacy of using TBL pedagogy in improving the teaching quality of various pharmacy curricula in China, however, the relevant systematic review has not been established. Therefore, the present meta-analysis was performed with the aim to evaluate systematically the effectiveness of TBL pedagogy adopted in Chinses pharmacy education. We hope that the present study can not only verify the availability of TBL in Chinese pharmacy education system, but also provide some useful information for other regions with similar pedagogical structures in China.

## Methods

This meta-analysis was conducted consistent with the recommendations in the Preferred Reporting Items for Systematic Reviews and Meta Analyses statement [12] and the guidelines described in the Cochrane Handbook.

### Search strategy

Two independent reviewers (BL and LH) performed the literature search. The databases included China National Knowledge Infrastructure (CNKI), Chinese VIP database, Chinese Wanfang Database, PubMed, Embase, and Cochrane Library were searched. The last literature search was performed on January 11, 2019 using the following search terms: “TBL”, “Team-based learning”, “pharmac*”, “pharmac* education”, “pharmac* students”. The details are shown in Additional file 1: Search strategy.

### Selection criteria

The studies under the following properties were enrolled:

- *Participants:* Students from Chinese pharmacy institutions.
- *Intervention:* Using TBL pedagogy in pharmacy curricula teaching.
- *Comparison:* Using traditional LBL in pharmacy curricula teaching.
- *Outcomes:* Theoretical test scores (Primary outcome); The incidence of students who accepted the effects of TBL pedagogy on improving their abilities (e.g. learning enthusiasm, self-study ability, thinking ability, and communication skills) from the questionnaires (Secondary outcome).
- *Study design:* Randomized controlled studies with no language limitations.

### Literature screening and data extraction

Literature screening and data extraction were carried out by two independent reviewers (BL and LH) and then they cross checked with each other. Full articles were obtained when details from titles and abstracts could not be clarified. The information about general characteristics of studies was collected in a designed table (Table 1). Disagreements were resolved by consensus through discussion among all authors.

**Table 1:** The general characteristics of the enrolled studies

Study ID	Year	Disciplines or curricula	Sample size (TBL/LBL)	Participant characteristics	Interventions	Comparisons	Outcome
Shang Z et al. [13]	2018	Pharmacotherapeutics	100 (50/50)	Clinical pharmacy students from University	Team-based Learning Details of teaching process: The responsibility of teachers including: laying out the discussion topic, raising the questions (or cases), organizing the post-tests and the questionnaires, and effectiveness evaluating. The responsibility of student groups: preview, information searching, literature reading, group discussion, and summary report.	Lecture	①②
Mao Y et al. [14]	2013	Drug evaluation and research	58 (28/30)	Pharmacy students from University	Team-based Learning Details of teaching process: The responsibility of teachers including: laying out the discussion topic, raising the questions, organizing the post-tests and the questionnaires, and effectiveness evaluating. The responsibility of student groups: Preview, information searching, literature reading, group discussion, and summary report.	Lecture	①
Feng X et al. [15]	2016	Pharmacy administration	123 (61/62)	Pharmacy students from University	Team-based Learning Details of teaching process: The responsibility of teachers including: laying out the discussion topic, organizing the pre-test (to evaluate the conditions of students’ preview), organizing the post-tests and the questionnaires, and effectiveness evaluating The responsibility of student groups: Preview, literature reading, group discussion, and summary report.	Lecture	①
Yan J et al. [16]	2016	Analytical chemistry	70 (35/35)	Pharmacy students from College	Team-based Learning Details of teaching process: The responsibility of teachers including: laying out the discussion topic, raising the questions, organizing the post-tests and the questionnaires, and effectiveness evaluating The responsibility of student groups: Preview, literature reading, group discussion, and summary report.	Lecture	①
Luo X et al. [17]	2017	Pharmaceutical chemistry	241 (124/117)	Pharmacy students from Secondary technical College	Team-based Learning Details of teaching process: The responsibility of teachers including: laying out the discussion topic, raising the questions, organizing the post-tests and the questionnaires, and effectiveness evaluating The responsibility of student groups: Preview, literature reading, group discussion, and summary report.	Lecture	①②
Gao M et al. [18]	2015	Pharmaceutics and experiments	48 (23/25)	pharmacy students from University	Team-based Learning Details of teaching process: The responsibility of teachers including: laying out the discussion topic, raising the questions, organizing the pre-test (to evaluate the conditions of students’ preview), organizing the post-tests and effectiveness evaluating The responsibility of student groups: preview, literature reading, group discussion for experiments operation, summarizing the points during experiments operation.	Lecture	①
Zhang H et al. [19]	2014	Medical functional experiments	50 (25/25)	pharmacy students from University	Team-based Learning Details of teaching process: The responsibility of teachers including: laying out the discussion topic, raising the questions, organizing the pre-test (to evaluate the conditions of students’ preview), organizing the post-tests and effectiveness evaluating. The responsibility of student groups: preview, literature reading, group discussion for experiments operation, summarizing the points during experiments operation.	Lecture	①
Xie Z et al. [20]	2015	Processing of traditional Chinese medicine	180 (90/90)	Pharmacy students from College	Team-based Learning Details of teaching process: The responsibility of teachers including: laying out the discussion topic, raising the questions, organizing the post-tests and effectiveness evaluating. The responsibility of student groups: preview, literature reading, group discussion for experiments operation, summarizing the points during experiments operation.	Lecture	①
Wu X et al. [21]	2017	Pharmacology	40 (20/20)	Oversea pharmacy students from University	Team-based Learning Details of teaching process: The responsibility of teachers including: laying out the discussion topic, raising the questions, organizing the pre-test (individual readiness assurance test), organizing the post-tests and effectiveness evaluating. The responsibility of student groups: preview, information searching, literature reading, group discussion, and summary report.	Lecture	①
Yang T et al. [22]	2018	Physical Chemistry	135 (63/72)	Pharmacy students from College	Team-based Learning Details of teaching process: The responsibility of teachers including: laying out the discussion topic, raising the questions, organizing the pre-test (to evaluate the conditions of students’ preview), organizing the post-tests and the questionnaires, and effectiveness evaluating. The responsibility of student groups: preview, information searching, literature reading, group discussion, and summary report.	Lecture	①
Yang X et al. [23]	2018	Medical biology experiments	64 (32/32)	Pharmacy students from University	Team-based Learning Details of teaching process: The responsibility of teachers including: laying out the discussion topic, raising the questions, organizing the post-tests and the questionnaires, and effectiveness evaluating. The responsibility of student groups: preview, literature reading, group discussion for experiments operation, summarizing the points during experiments operation.	Lecture	①②
Yi T et al. [24]	2018	Biochemistry	90 (45/45)	Pharmacy students from University	Team-based Learning Details of teaching process: The responsibility of teachers including: laying out the discussion topic, raising the questions, organizing the pre-test (to evaluate the conditions of students’ preview), organizing the post-tests and the questionnaires, and effectiveness evaluating. The responsibility of student groups: preview, information searching, literature reading, group discussion, and summary report.	Lecture	①②

Notes:① Theoretical tests scores; ② Questionnaires for assessing the effects of pedagogies on students’ qualities and abilitie

### Quality assessment

Following the Cochrane Collaboration tool for assessing risk of bias [25], two reviewers (BL and LH) independently evaluated the methodological quality of the enrolled studies. Seven items including allocation concealment, blinding of participants and personnel, blinding of outcome assessment, incomplete outcome data, selective reporting and other bias were checked and graded by high, low, or unclear risk of bias [26].

### Statistics analysis

Statistical analyses were conducted with Review Manager 5.0 software (The Cochrane Collaboration, London, UK). The risk ratio (RR) with 95% CI and the Mantel–Haenszel method (fixed or random models) were used to analyze dichotomous data (such as the outcome of questionnaires for assessing the effects of pedagogies on students’ qualities and abilities); and for continuous data (for instance, the tests scores), standardized mean difference (SMD) was chosen for the estimation. The impact of heterogeneity on results was weighed by using the *I*-squared (*I*^2^) test. According to Cochrane review guidelines, the fixed-effects model was employed to pool data if there was no heterogeneity (*I*^2^ < 50%); Otherwise, the random-effects model was adopted when severe heterogeneity was present at *I*^2^ > 50% (or the value of *I*^2^ was closed to 50%). Then, sensitivity analysis was performed by excluding each study individually to reassess the quality and consistency of the results. Subgroup analyses were used to explore the diversity among different studies and source of heterogeneity, and we used two variables: students from colleges (3-year program) versus students from universities (4-year program) and experiments-oriented courses versus theory-oriented courses. And we used Begg’s test and Egger’s test to evaluate the publication bias when at least ten studies were enrolled in analysis. A *p* value less than 0.05 was deemed to be statistically significant.

## Results

### Literature search results

**Fig. 1:**
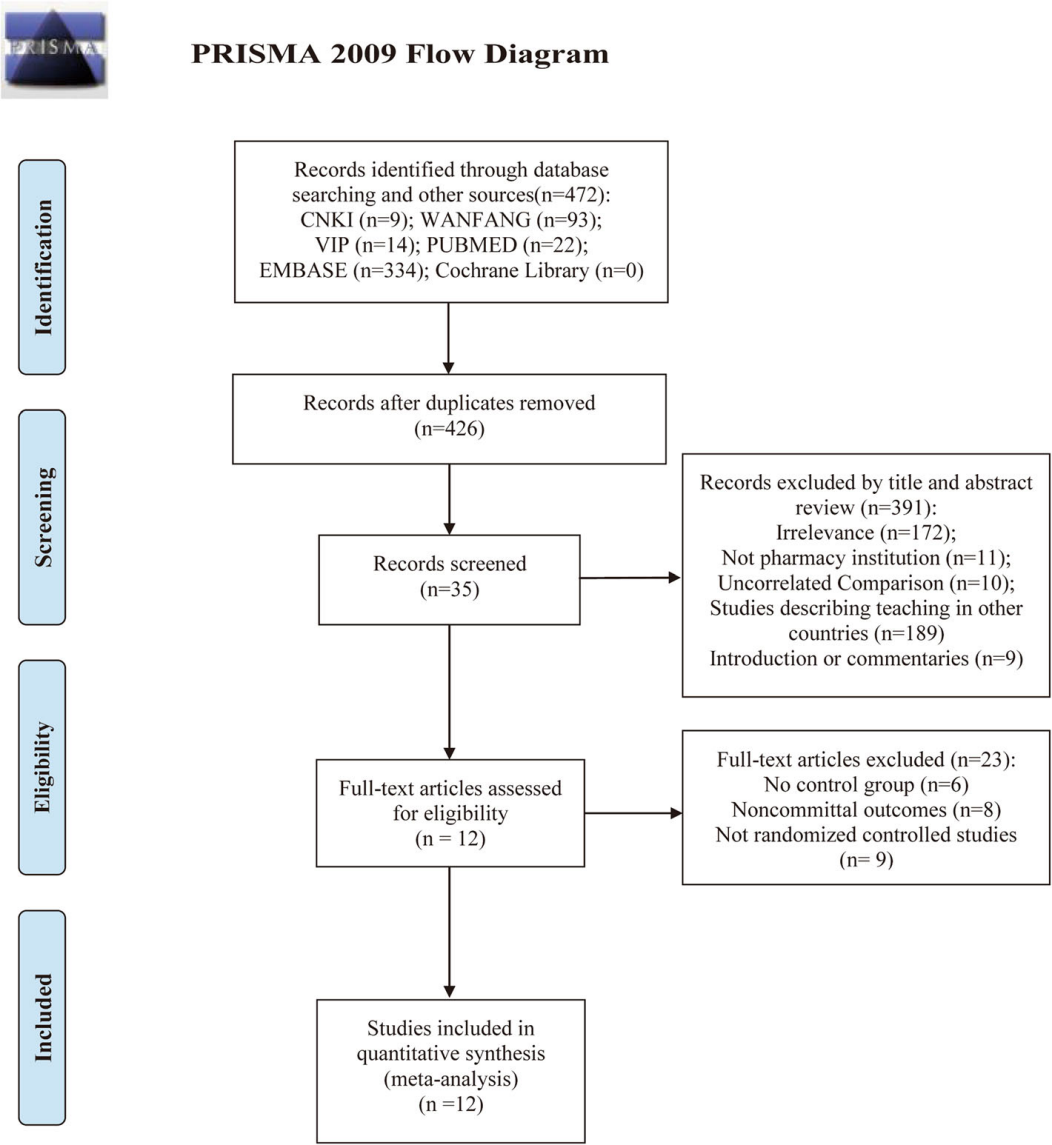
Flow chart of literature screening and the selection process

After searching of databases, 472 items were identified, and 46 of them were excluded by duplicate removing. Of 391 articles excluding in title and abstract review, 172 were not related to theme of our present study, 11 were not performed in the pharmacy institutions, 189 were not performed in mainland of China, 10 were focused the uncorrelated comparison (for instance, the comparison between using the combination of TBL and Case-based learning pedagogy versus using only Case-based learning pedagogy), and 9 were introductions or commentaries. After full-text review, 23 articles were excluded (6 were owing to the absence of control group, 8 were on account of the noncommittal outcome, and 9 of them were not randomized controlled studies. Eventually, 12 studies were enrolled in further analysis [13–24]. The procedure of eligible articles screen is described in Fig. 1.

### Basic characteristics of enrolled studies

The included studies were published from 2013 to 2018 in Chinese. And the relevant pharmacy curricula including: Pharmacotherapeutics, Drug evaluation and research, Pharmacy administration, Analytical chemistry, Pharmaceutical chemistry, Pharmaceutic experiments, Medical functional experiments, Processing of traditional Chinese medicine, Pharmacology, Physical Chemistry, Medical biology experiments and Biochemistry. All studies reported the outcome of test scores for evaluating the effectiveness of two pedagogy methods. The questionnaires to assess students’ qualities and abilities were performed in four studies [13, 17, 23, 24], all of them mentioned the effects of pedagogies on developing students’ learning enthusiasm and self-study ability, 3 of them mentioned their effects on developing thinking ability, and 2 studies described their effects on enhancing communication skills. The general characteristics of the enrolled studies are shown in Table 1.

### Quality assessment

**Fig. 2:**
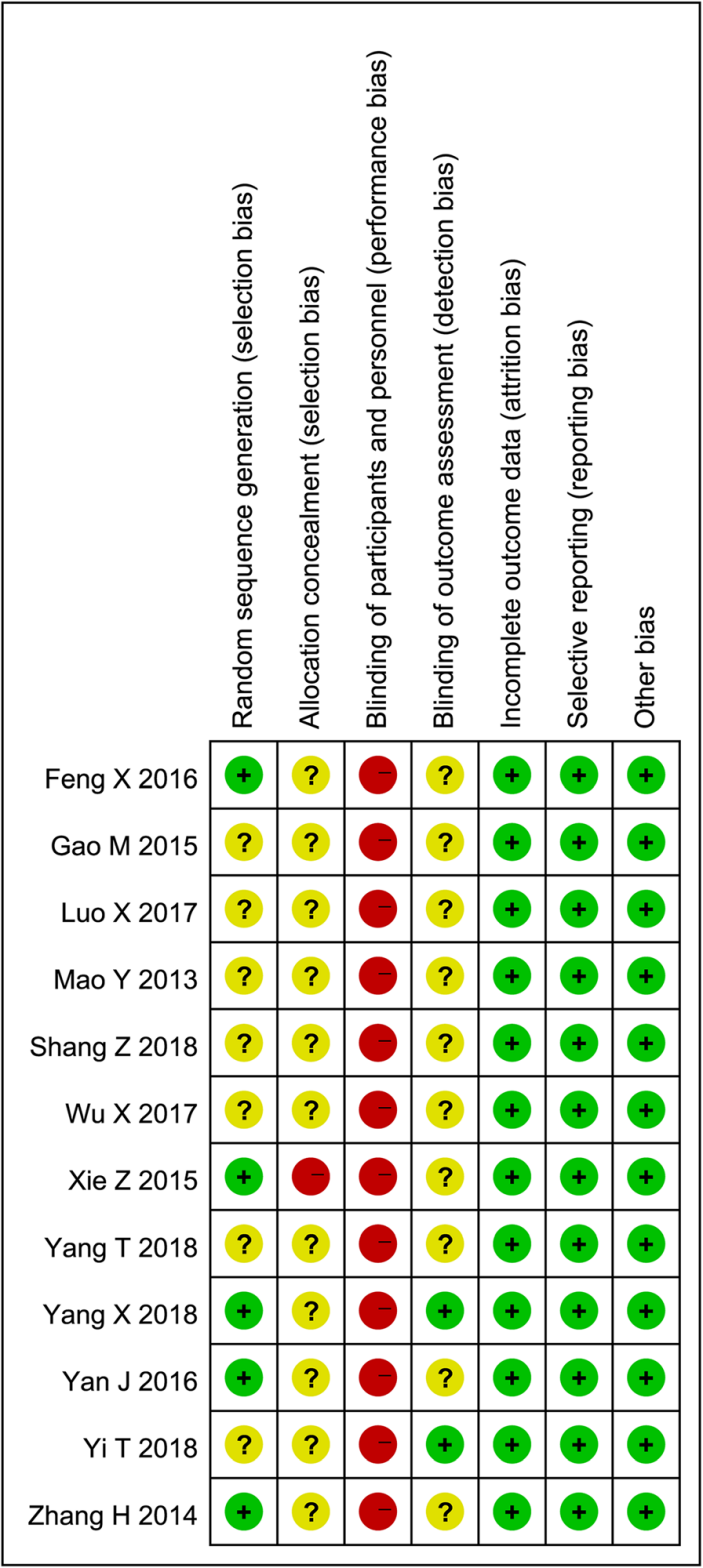
Risk of bias assessment of included studies. Notes: Green + dot, low risk of bias; yellow? dot, unclear risk of bias; red - dot, high risk of bias

Allocation concealment, blinding of participants and personnel, blinding of outcome assessment, incomplete outcome data, selective reporting and other bias were evaluated in accordance with Cochrane Collaboration tool for assessing risk of bias. 40% (5/12) of studies employed an adequate method of random sequence generation [15, 16, 19, 20, 23], and only two studies reported blinding procedure of outcome assessment [23, 24]. On account of the characteristics of two pedagogy methods, the participants and personnel could not be blinded in any of these studies. The overview of risk of bias assessment are shown in Fig. 2.

### Effects of two pedagogies on theoretical scores

**Fig. 3:**
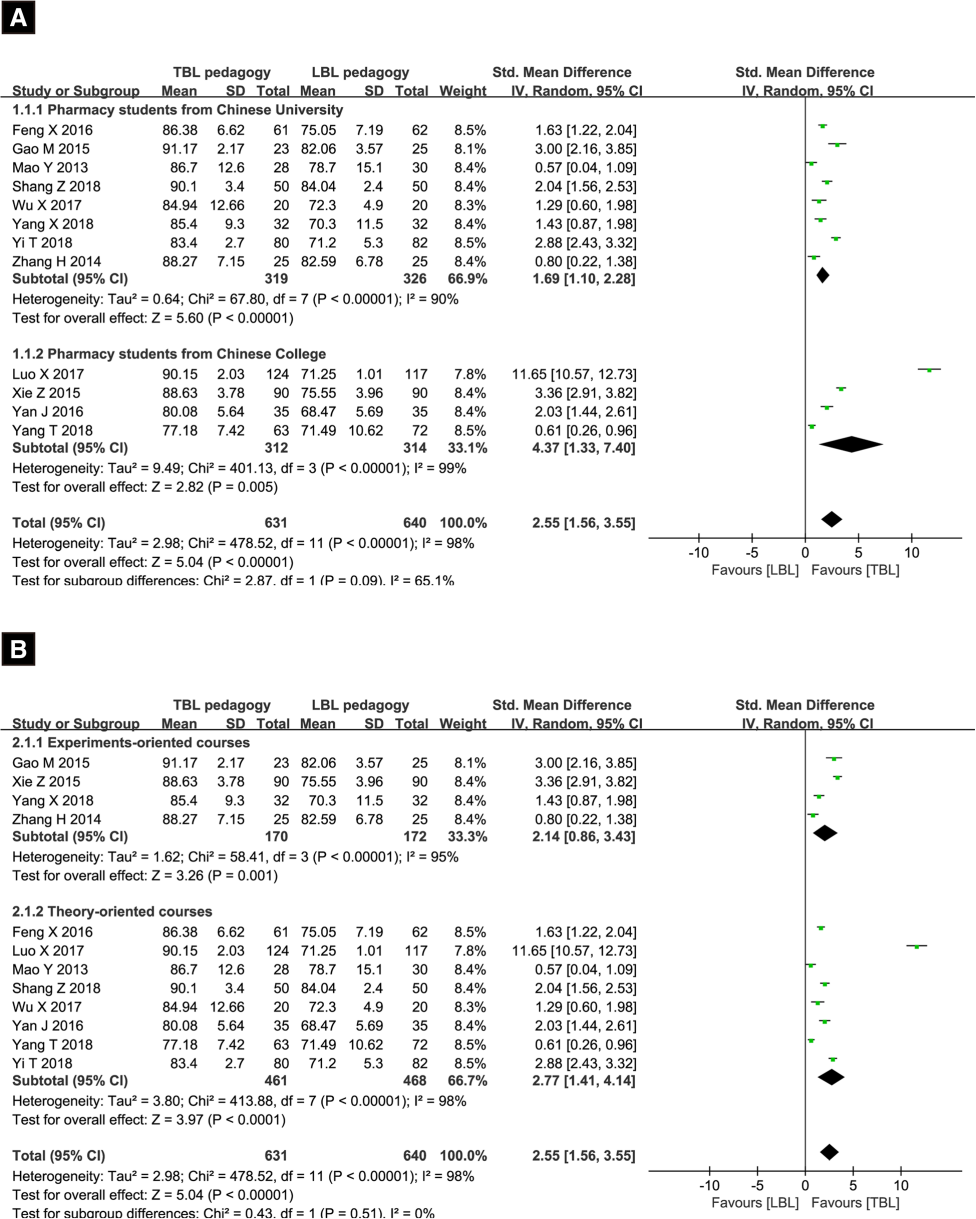
Forest plot of theoretical scores for TBL compared with LBL. (**a)** Subgroup analysis of scores of students from colleges versus students from universities; (**b**) Subgroup analysis of scores of students experienced experiments-oriented courses versus theory-oriented courses. Squares symbolize SMD point estimate, and horizontal lines represent 95% CI. The diamond represents the overall summary estimate. (CI: confidence interval; SMD: standardized mean difference)

**Fig. 4:**
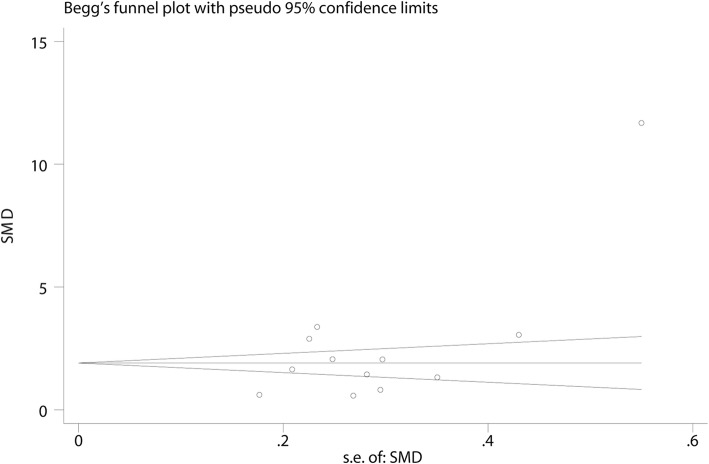
Begg’s Funnel plots of theoretical scores with pseudo 95% confidence limits (Theoretical scores for TBL compared with LBL. Egger’s test, *p* = 0.049; Begg’s Test, *p* = 0.373)

All studies involving 1271 participants were included, 631 of them were receiving the TBL pedagogy in various pharmacy curricula learning. Owing to the existence of heterogeneity (*I*^*2*^ = 98%), the random-effects model was chosen. Compared with the ones receiving traditional LBL pedagogy, students receiving TBL pedagogy were tend to get higher marks in objective theoretical tests (SMD = 2.55, 95% CI [1.56, 3.55], *p* < 0.00001). For the substantial heterogeneity between studies was observed, the sensitivity analysis was performed to reevaluate the quality and consistency of the results by omitting individual study sequentially. However, the source could not be clearly attributed to a single study. The results from subgroup analyses including two variables (different education background and different types of curricula) indicated that TBL pedagogy was beneficial to both students who experienced 4-year program education from universities (SMD = 1.69, 95% CI [1.10, 2.28], *p* < 0.00001) and the ones who received 3-year program education from colleges (SMD = 4.37, 95% CI [1.33, 7.40], *p* < 0.00001), and TBL pedagogy also applied well in experiments-oriented courses (SMD = 2.14, 95% CI [0.86, 3.43], *p* < 0.00001) and theory-oriented courses (SMD = 2.77, 95% CI [1.41, 4.14], *p* < 0.00001). However, high level of *I*^*2*^ in subgroup analyses indicated the heterogeneity still existed. The results are shown in Fig. 3a and b. The results of Egger’s (*p* = 0.049) tests indicated publication bias was existed (Fig. 4). Then the Duval’s trim and fill method was performed to estimate and adjust for the number and outcomes of missing studies [27]. Although the existence of publication bias, results from sensitivity analyses of trim and fill method (no new studies added) exhibited that the result was reliable.

### The incidence of students who accepted the effects of TBL pedagogy on improving their abilities

#### Effects on developing students’ learning enthusiasm

**Fig. 5:**
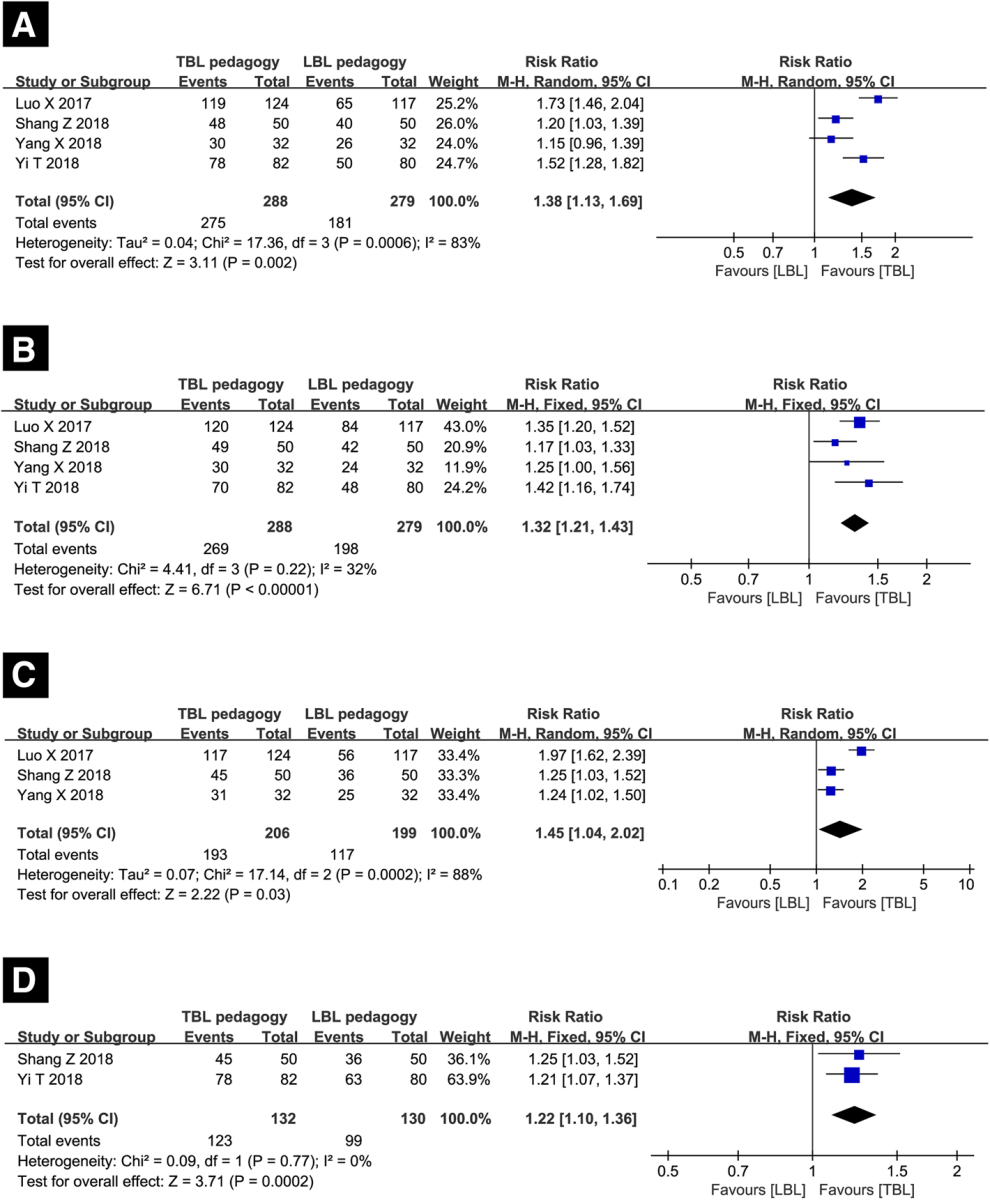
Forest plot of the students’ various qualities and abilities for TBL compared with LBL. (**a)** Effects on developing students’ learning enthusiasm; (**b**) Effects on developing students’ self-study ability; (**c**) Effects on developing students’ thinking ability; (**d**) Effects on enhancing students’ communication skills. The explanation for forest plot is stated in Fig. 3

Four studies involving 567 participants were analyzed, and 288 of them were receiving TBL pedagogy. Compared with the application of traditional LBL pedagogy method, the results from questionnaires indicated that the introduction of TBL pedagogy developed more students’ learning enthusiasm (the ratio of students thinking the pedagogy method had great influence on developing learning enthusiasm: 95.49% in TBL group vs 64.87% in LBL group, RR = 1.38 with 95% CI [1.13,1.69], *p* < 0.0001, *I*^2^ = 83%). The substantial heterogeneity cannot be resolved by deleting a single study, thus, the random effect was performed (Fig. 5a).

#### Effects on developing students’ self-study ability

A total of 567 students from four studies were included, and 288 of them were receiving TBL pedagogy. The results from questionnaires indicated that the introduction of TBL pedagogy also developed more students’ self-study ability compared to LBL (the ratio of students thinking the pedagogy method had great influence on developing self-study ability: 93.40% in TBL group vs 70.97% in LBL group, RR = 1.32 with 95% CI [1.21,1.43], *p* < 0.0001, *I*^2^ = 32%). The statistical heterogeneity did not exist among the study results; thus, a fixed-effects model was employed to perform the analysis (Fig. 5b).

#### Effects on developing students’ thinking ability

Three studies involving a total of 405 students were enrolled, and 206 of them were receiving TBL pedagogy method. The responses of questionnaires revealed that the application of TBL significantly developed students’ thinking ability compared to LBL (the ratio of students thinking the pedagogy method had great influence on developing thinking ability: 93.69% in TBL group vs 58.79% in LBL group, RR = 1.45 with 95% CI [1.04,2.02], *p* < 0.0001, *I*^2^ = 88%). The heterogeneity was resolved after removing the Luo study [17] (*I*^2^ = 0%), and the summary estimate was unchanged essentially (the ratio of students thinking the pedagogy method had great influence on developing thinking ability: 92.68% in TBL group vs 74.39% in LBL group, RR = 1.24 with 95% CI [1.08,1.43], *p* < 0.0001). The result is shown in Fig. 5c.

#### Effects on enhancing students’ communication skills

Two studies involving 262 students were analyzed, and 132 of them were receiving TBL method. The *I*^2^ of 0% indicated that there was no substantial heterogeneity, thus the fixed effect model was chosen. The results from the questionnaires demonstrated that TBL method enhanced students’ communication skills obviously (the ratio of students thinking the pedagogy method had great influence on enhancing communication skills: 93.18% in TBL group vs 76.15% in LBL group, RR = 1.22 with 95% CI [1.10,1.36], *p* < 0.0001). The result is shown in Fig. 5d.

## Discussion

For restrictions of traditional teaching conceptions, the lecture-based learning was dominated in Chinese education structure. Nevertheless, the condition of domestic education has been changed as the introduction of the innovative pedagogical approaches over the recent years. For instance, problem-based learning (PBL) was an educational innovation developed in recent decades especially in China. Many Chinese pharmacy institutions have made tentative steps in practicing such novel pedagogy. Zhou et al. have performed the analysis systematically to verify the effectiveness of such novel pedagogy methods in domestic pharmacy education [10]. As a pedagogical model of small-group learning, TBL also has been approved and highly praised in health science education [28] for its efficiency in improving teaching quality and for its high satisfaction in students. The effectiveness of TBL pedagogy was also supported by similar studies performed in some developed countries, and the scope of its application covered medical, nursing, and pharmacy education [29–31]. Compared with the application of TBL in Chinese medical education [9], it started even later in pharmacy education of China. The present study was the first meta-analysis to evaluate the effectiveness of TBL on Chinese pharmacy education. We also would like to explore more useful information about the applicability of such pedagogical method in domestic educational institutions at different levels and in different types of curricula (pure theory-oriented courses/experiments-oriented courses). In addition, we expected to learn which aspects of students’ quality would be improved by application of TBL.

By summarizing the relevant studies about the application of TBL in Chinese pharmacy education, the present study demonstrated that such pedagogy has been introduced and even applied widely during last decade in China. The time range of published literatures (2013–2018) means it is still in an embryonic stage, but it has served Chinese pharmacy education well in various institutions at different hierarchical level. We are encouraged by the results of analysis that students receiving TBL process gained higher scores in theoretical tests compared to the students receiving traditional LBL process. Considering the substantial heterogeneity, we performed sensitivity analysis and chose the random-effects model, and the positive effects were also verified.

To assess the effects of two pedagogies on students’ qualities and abilities, according to the descriptions in some articles, the questionnaires were sent to the participants. The ratio of students thinking the pedagogy method had great influence on developing various qualities and abilities was analyzed in our study. We obtained more positive feedbacks from the students who receiving TBL pedagogy, and they were manifested by the developments of students’ learning enthusiasm, self-study ability, thinking ability, and the enhance of students’ communication skills.

Chinese pharmacy institutions are divided into different levels. Compared with the students from colleges (3-year program), the students from universities (4-year program) have higher scores of entrance examination and stronger continuous learning capability. Remarkably, the results of present study indicated that both the students from universities and those from colleges experienced considerable developments after receiving TBL teaching method, which represents the adaptability of TBL pedagogy in Chinese pharmacy education. It also appears that such pedagogy should be generalized in more Chinese classrooms.

### Limitations and future studies

The current research’s limitations derived from the methodological quality and design of the enrolled studies. Firstly, owing to the open environment of teaching process, allocation concealment and blinding of participants were difficult to be practiced. Secondly, as the search strategies were strictly delimited to the areas in Chinese classrooms, plenty of literatures published in Chinese were analyzed in present research. The results of Egger’s (*p* = 0.049) tests indicated the existence of publication bias, however, there was no indications of publication with the trim and fill method (no new studies added), which demonstrated that the result was reliable. Furthermore, to explore the reasons for substantial the heterogeneity found in the main analysis, subgroup analyses were conducted for two variables (1. Scores of students from colleges versus students from universities; 2. Scores of students experienced experiments-oriented courses versus theory-oriented courses). However, the major cause of heterogeneity is still uncertain. The source of heterogeneity may be resulted from unreported factors (e.g. teaching resources).

In addition, the published literatures indicated that the number of methods to evaluate the effectiveness of pedagogy were limited. We collected and analyzed two sorts of outcomes including the theoretical scores and the responses of questionnaires. However, there were different types of questionnaires during data collection, thus, we performed data combination only for the studies containing the same items, and the effects investigation of TBL pedagogy focuses on the following four aspects: learning enthusiasm, self-study ability, thinking ability, and communication skills of students. Actually, to comprehensive evaluate the effectiveness of TBL pedagogy in depth, high-quality evidences with multiple and standardized evaluation methods are necessary.

## Conclusion

The results of our study indicated the gratifying effectiveness of TBL application in Chinese pharmacy institution during last decade. It enhanced the theoretical scores and various abilities of Chinese pharmacy students. To evaluate the effectiveness of TBL pedagogy comprehensively, it calls for more high-quality studies and more standardized evaluation methods in future. Overall, TBL pedagogy is compatible with the present Chinese pharmacy education, and it should be generalized in more classrooms.

## Additional file

Additional file 1: Search Strategy. Details of search strategy in different databases. (DOCX 16 kb)

## Data Availability

The data analyzed in our study were collected from available published articles.

## Abbreviations

CI: Confidence interval
CNKI: Chinese national knowledge infrastructure
LBL: Lecture-based learning
RR: Risk Ratio
SMD: Standardized mean difference
TBL: Team-based learning
