# Supplementary material for: Team-based learning pedagogy enhances the quality of Chinese pharmacy education: a systematic review and meta-analysis
Source: BMC Med Educ. 2019 Jul 29;19:286. doi: 10.1186/s12909-019-1724-6 (PMC6664710; doi:10.1186/s12909-019-1724-6)
Supplement: Supplementary file 1 — Search Strategy. Details of search strategy in different databases. (DOCX 16 kb) [file 12909_2019_1724_MOESM1_ESM.docx]

PUBMED:

| Search | Query |
| --- | --- |
| #1 | TBL OR "Team-based learning" |
| #2 | ("pharmac*" OR "pharmac* education" OR "pharmac* students") |
| #3 | #1 AND #2 |
| Items found: 22 | |

EMBASE:

| Search | Query |
| --- | --- |
| 1 | TBL OR 'Team-based learning' |
| 2 | 'pharmac*' OR 'pharmac* education' OR 'pharmac* students' |
| 3 | 1 and 2 |
| Items found: 334 | |

Cochrane Library:

| Search | Query |
| --- | --- |
| #1 | TBL OR "Team-based learning" |
| #2 | ("pharmac*" OR "pharmac* education" OR "pharmac* students") |
| #3 | #1 AND #2 |
| Items found: 0 | |

CNKI

| Search | Query |
| --- | --- |
| #1 | ((TI='TBL' OR KY='TBL') OR (TI='Team-based learning' OR KY='Team-based learning')) |
| #2 | (TI='Pharmacy' in Chinese OR KY='Pharmacy' in Chinese) |
| #3 | #1 AND #2 |
| Items found: 9 | |

WANFANG:

| Search | Query |
| --- | --- |
| #1 | ((TI='TBL' OR KY='TBL') OR (TI='Team-based learning' OR KY='Team-based learning')) |
| #2 | (TI='Pharmacy' in Chinese OR KY='Pharmacy' in Chinese) |
| #3 | #1 AND #2 |
| Items found: 93 | |

Chinese VIP:

| Search | Query |
| --- | --- |
| #1 | ((TI='TBL' OR KY='TBL') OR (TI='Team-based learning' OR KY='Team-based learning')) |
| #2 | (TI='Pharmacy' in Chinese OR KY='Pharmacy' in Chinese) |
| #3 | #1 AND #2 |
| Items found: 14 | |
